# The shedded ectodomain of Lyve-1 expressed on M2-like tumor-associated macrophages inhibits melanoma cell proliferation

**DOI:** 10.18632/oncotarget.21771

**Published:** 2017-10-10

**Authors:** Claudia Dollt, Kathrin Becker, Julia Michel, Susanne Melchers, Cleo-Aron Weis, Kai Schledzewski, Andreas Krewer, Loreen Kloss, Christoffer Gebhardt, Jochen Utikal, Astrid Schmieder

**Affiliations:** ^1^ Department of Dermatology, Venereology and Allergology, University Medical Center and Medical Faculty Mannheim, University of Heidelberg, and Center of Excellence in Dermatology, Mannheim, Germany; ^2^ Department of Innate Immunity and Tolerance, Institute of Transfusion Medicine and Immunology, University Medical Center and Medical Faculty Mannheim, University of Heidelberg, Mannheim, Germany; ^3^ Institute of Pathology, University Medical Center and Medical Faculty Mannheim, University of Heidelberg, Mannheim, Germany; ^4^ Skin Cancer Unit, German Cancer Research Center (DKFZ), Heidelberg, Germany

**Keywords:** M2-like macrophages, tumor-associated macrophages, Lyve-1, melanoma, decoy receptor

## Abstract

Targeting immune cells that support tumor growth is an effective therapeutic strategy in tumor entities such as melanoma. M2-like tumor-associated macrophages (TAM) sustain tumor growth by secreting anti-inflammatory cytokines, proteases and growth factors. In this study, we show that a protein derived from M2-like macrophages namely the shedded ectodomain of Lyve-1 (sLyve-1) decreases human HT144 and murine B16F1 melanoma cell proliferation significantly by acting as a decoy receptor for low-molecular weight hyaluronic acid (LMW-HA) although the LMW-HA/Lyve-1 interaction on lymphatic endothelial cells has been described to induce lymphangiogenesis. This is in line with our finding that the number of LYVE-1^+^ TAM decreases in higher human melanoma stages and that the early growth of B16 transplant tumors is enhanced in *Lyve-1* knockout mice when compared to wild-type mice due to an increased melanoma cell proliferation. LYVE-1 expressing TAM are however true M2 macrophages as they co-express typical M2-markers such as CD163 and CD206. The results of the present study highlight the necessity to carefully determine the net effect particular TAM subpopulations have on tumors before establishing a treatment to target these immune cells.

## INTRODUCTION

Hyaluronic acid (HA) is a structural component of the extracellular matrix necessary for hydration and protection against mechanical forces. It binds to a wide range of different receptors - amongst them the lymphatic vessel endothelial hyaluronan receptor-1 (Lyve-1) - and exerts various functions which also depend on the size of the polymer. While high molecular weight-HA (HMW-HA) for example has an anti-inflammatory effect, low molecular weight-HA (LMW-HA) promotes inflammation [1, 2]. Concerning tumor growth, excessive production of HA due to upregulated expression of HA-synthases is associated with the formation of aggressive tumors. High HA levels further correlate with tumor progression in different tumor entities, such as breast, ovarian, prostate and colorectal cancer [3, 4].

Lyve-1 is a type I transmembrane glycoprotein belonging to the link domain superfamily with 43 % sequence homology to the hyaluronan receptor CD44. It has first been identified as a lymphatic endothelial cell-specific HA receptor [5-7]. Lyve-1 receptor engagement by LMW-HA in lymphatic endothelial cells promotes cell proliferation, migration and thus lymphangiogenesis [8, 9]. However, LYVE-1 has also been found to be expressed by other cell types, such as Kupffer cells [10] and tumor-associated macrophages (TAMs) [11]. The biological effect of a HA/Lyve-1-binding in these myeloid cells has not yet been examined.

In this study, we analyzed the possible implication of LMW-HA binding to soluble Lyve-1 derived from TAMs on tumor growth. TAMs have been described to exert important tumor-supportive roles by providing angiogenic, anti-inflammatory and matrix-remodelling factors, characteristics typically displayed by alternatively activated or M2-like macrophages [12, 13].

We found that macrophage-derived shedded Lyve-1 was able to inhibit LMW-HA-dependent proliferation of human and murine melanoma cells, pointing towards a possible decoy receptor function of the shedded receptor. LMW-HA/Lyve-1 interaction is therefore a two-edged sword for tumors such as melanoma as on the one hand it promotes lymphangiogenesis and therefore lymphangiogenic metastasis; on the other hand it inhibits LMW-HA dependent melanoma cell proliferation.

## RESULTS

### The number of LYVE-1^+^ macrophages decreases in melanoma metastasis

**Figure 1:**
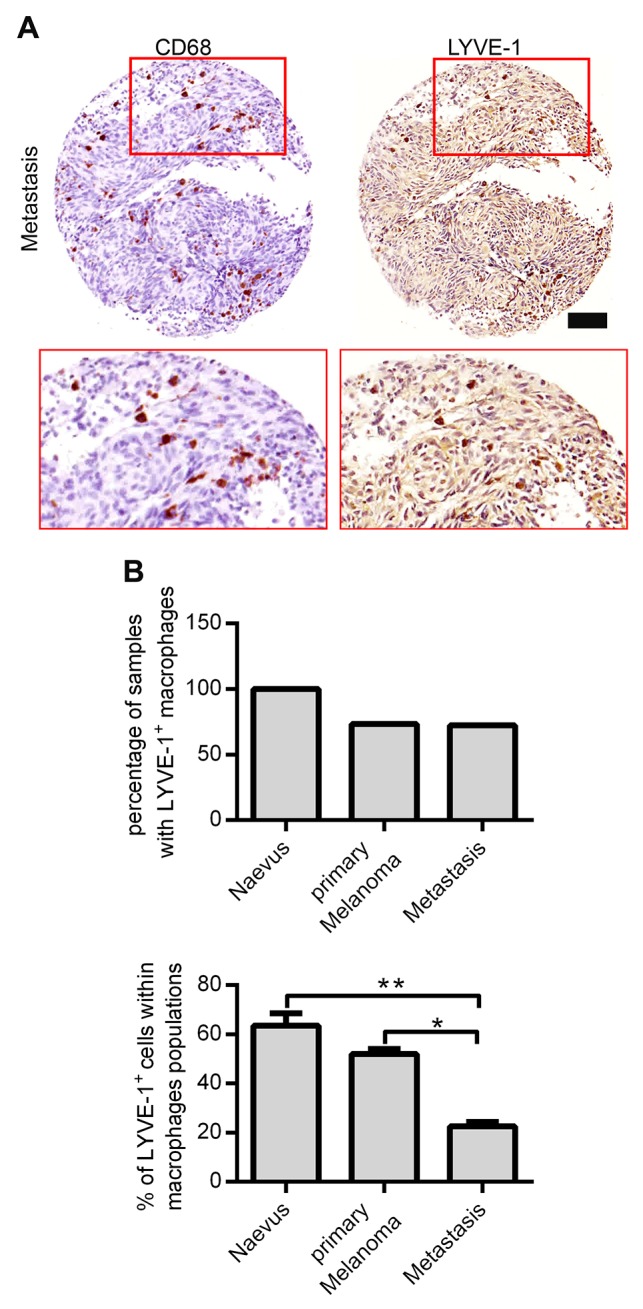
Identification and quantification of Lyve-1^+^ macrophages in benign and malignant melanocytic lesions **(A)** Two tissue microarrays with 105 specimens of benign and malignant melanocytic lesions in duplicates were sequentially stained with a CD68-antibody (red) and, after a destaining procedure, again with an anti-Lyve-1 antibody (brown). One representative specimen of a metastatic melanocytic lesion is depicted, scale bar = 100 μM. **(B)** Quantification of CD68^+^ and LYVE-1^+^ cells in sequentially stained TMA. Two core sections of each melanocytic lesion were evaluated after sequential immunohistochemical staining. First, the total number of sections containing LYVE-1^+^ macrophages was enumerated (upper panel), thereafter, the percentage of LYVE-1^+^ cells within the macrophage population was assessed per core section (bottom panel). Results are depicted as mean values plus SEM.

Previously, our group has identified LYVE-1^+^ TAMs in murine and human melanoma. However, this distinct macrophage subpopulation has neither been further quantified nor phenotypically and functionally been characterized. By sequential staining, which enables the staining of the same histopathological slide with different antibodies, we were able to analyze two tissue microarrays (TMA) containing melanocytic lesions with CD68 and LYVE-1 antibodies (Figure 1A). The TMAs were composed of 36 naevi, 27 primary melanoma, and 42 metastasis samples of which 17 naevi, 19 primary melanomas, and 29 melanoma metastasis were found to contain CD68^+^ macrophages (TMA described in [14]). The sequential staining of these samples revealed that in all naevi samples LYVE-1^+^ macrophages were present, while only in 73.3 % of primary melanoma samples and in 72.4 % of the metastasis CD68/LYVE-1 double positive cells were spotted (Figure 1B, upper panel). Moreover, the percentage of LYVE-1^+^/CD68^+^ double positive cells in relation to the total amount of CD68^+^ cells was assessed. In metastatic lesions significantly smaller portions of macrophages were LYVE-1^+^ compared to naevi and primary melanoma indicating a loss of LYVE-1^+^ TAM in higher melanoma stages (Figure 1B, lower panel).

### Melanoma growth is enhanced in *Lyve-1* knockout mice

**Figure 2:**
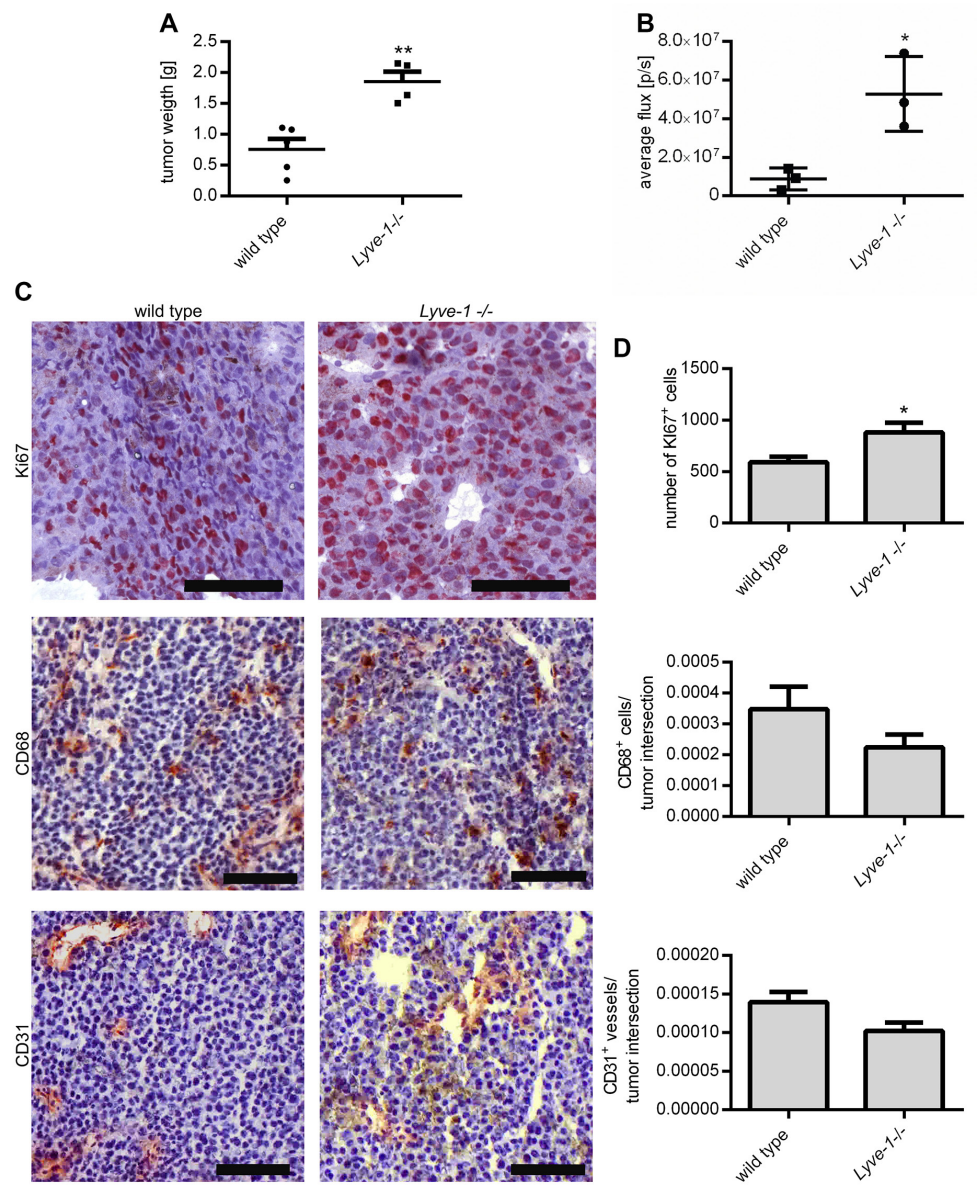
*In vivo Lyve-1* deficiency leads to increased tumor growth 1× 10^6^ Melanoma cells were injected subcutaneously into the flank of the C57BL/6 *Lyve-1*^-/-^ and wild type mice respectively. **(A)** Tumor end weight was determined ten days after the injection of B16F1, (n=9). **(B)** Monitoring of tumor growth by luminescence measurement following luciferin injection seven days after injection of B16F10 LUC, (n = 3). **(C)** Cryosections of B16F1 transplant tumors grown for ten days were stained with Ki67-, CD68- and CD31-antibody. Representative pictures are shown, scale bars=100 μm. **(D)** The number of Ki67^+^ cells was enumerated in five representative pictures per immunohistochemically stained tumor sample and the number of CD68^+^ cells and CD31^+^ vessels was assessed in relation to the area of whole tumor intersections, (n = 7). Results are depicted as mean values with SEM.

To determine the relevance of LYVE-1 on melanoma growth in general, a murine model with a global knockout of the *Lyve-1* gene was used. These mice have already been characterized elsewhere to have an overtly normal phenotype, developing a functional lymphoid system except for enlarged lymphatic vessels in the liver and intestine leading to a constitutively increased interstitial fluid flow [15, 16]. At first, the growth of B16F1 melanoma transplant tumors in C57BL/6 *Lyve-1* knockout mice was examined and compared to tumor growth in wild-type control mice. Ten days after subcutaneous injection of the melanoma cells, the mice were sacrificed and the weight of the excised non-ulcerated tumors was determined. *Lyve-1* deficiency led to a significantly increased tumor end weight (Figure 2A). Monitoring of B16F10 LUC tumors *in vivo* by luminescence measurement further sustained the tumor-inhibiting role of LYVE-1. Here, the average photon flux, which strongly correlates with tumor volume, was significantly increased in tumors developing in *Lyve-1* ^-/-^ mice already seven days after tumor cell injection compared to tumors in wild-type mice (Figure 2B). Immunohistological stainings of murine B16F1 transplant tumors revealed that significantly more cells were stained positive for the proliferation marker Ki-67 in tumors grown in C57BL/6 *Lyve-1* ^-/-^ correlating with the increased tumor end weight. In contrast, staining with antibodies against CD68 and CD31 revealed in both cases only minor, non-significant reductions of myeloid cells and vessels in tumors grown in the *Lyve-1* knockout mice (Figure 2C, 2D).

### LYVE-1 expression is associated with a M2-like macrophage phenotype

**Figure 3:**
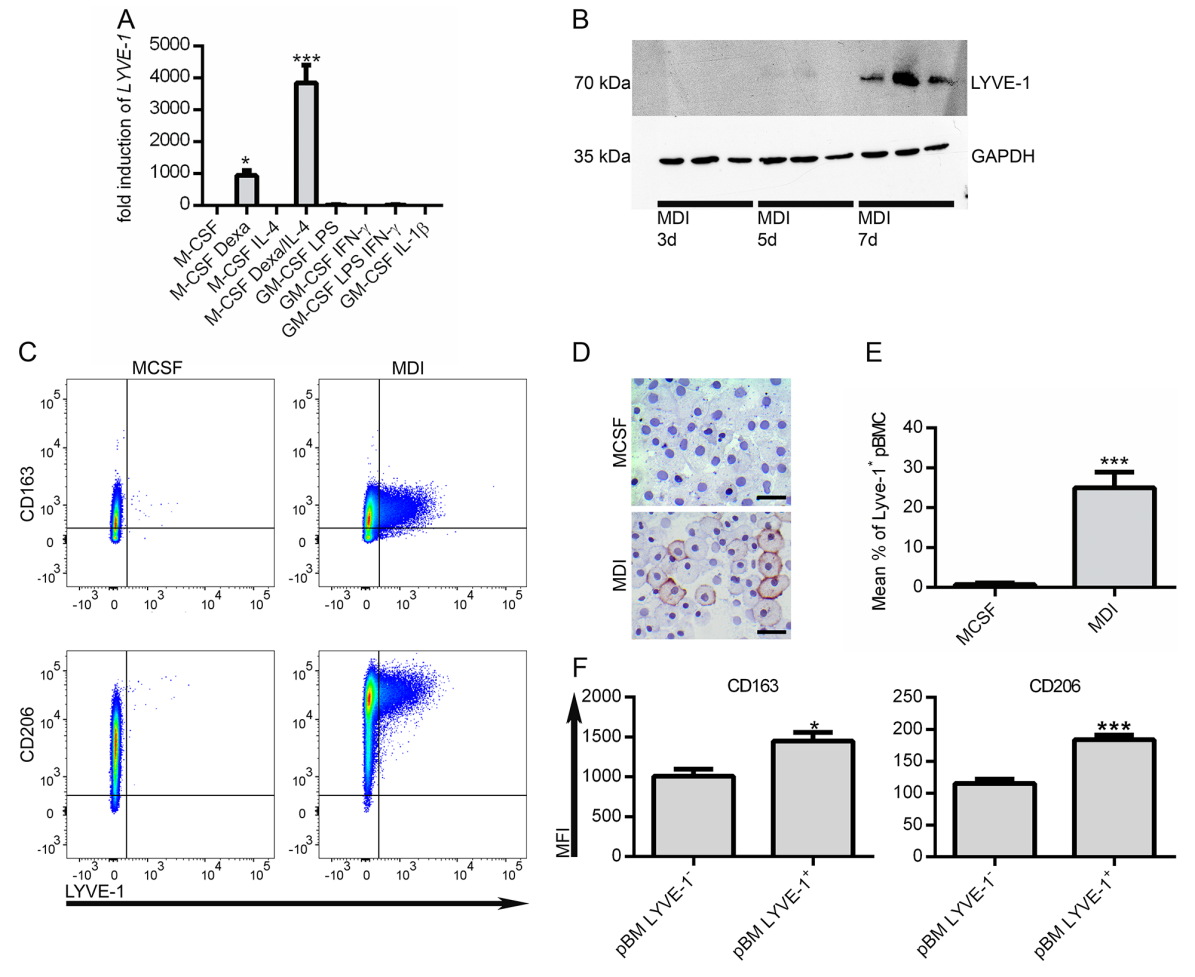
Lyve-1 is expressed in M2-like macrophages **(A)** Peripheral blood monocytes (pBM) were treated for seven days with different pro- and anti-inflammatory stimuli as indicated and *LYVE-1* expression was assessed as fold change over M-CSF treated pBM (n=3). **(B)** pBM were treated with MCSF/dexa/IL-4 (MDI) for 3, 5 and 7 days as indicated. Protein expression of LYVE-1 was determined by western blot (n=3). **(C)** Determination of co-expression of LYVE-1 with macrophage markers CD163 and CD206 respectively by FACS. Representative experiments are shown. **(D)** pBM were stimulated for seven days with M-CSF or MDI as indicated. Cytospins were fixed with PFA and stained with anti-LYVE-1, scale bars = 20 μM. **(E)** Flow cytometric quantification of LYVE-1 surface expression in M-CSF or MDI treated cells, (n = 8). **(F)** Comparison of marker expression levels between LYVE-1^+^ and LYVE-1^-^ MDI treated pBM by evaluation of MFI (median fluorescence intensity), (n = 10).

The aim of this study was to specifically analyze the possible relevance of LYVE-1 expression on TAMs for melanoma growth. *In vitro*, *Lyve-1* expression could be induced in murine bone marrow-derived macrophages (BMDM) by stimulation with B16F1 derived tumor-conditioned medium (TCM) in combination with the synthetic glucocorticoid dexamethasone (dexa) and interleukine-4 (IL-4), which have both been described to shape an alternative macrophage phenotype [11]. To transfer this to a human setting, peripheral blood monocytes (pBM) were stimulated with different pro- and anti-inflammatory stimuli. While macrophage-colony stimulating factor (M-CSF) in combination with dexa was sufficient to induce *LYVE-1* mRNA expression significantly, the addition of IL-4 to the stimulation cocktail led to a considerable boost of this effect. Pro-inflammatory stimuli did not lead to *LYVE-1* expression in pBM (Figure 3A). On protein level, LYVE-1 was detectable in a time-dependent manner after seven days of stimulation with M-CSF, dexa, and IL-4 (MDI) (Figure 3B).

To determine the activation status of the MDI-induced macrophages, expression levels of mannose receptor CD206 and scavenger receptor CD163, which have both been associated with a M2-like phenotype, were evaluated. Besides the induction of LYVE-1, MDI stimulation promotes the expression of both examined M2-markers on mRNA and protein level (Supplementary Figure 1). LYVE-1 was found to be co-expressed with the two examined macrophage markers (Figure 3C). Furthermore, LYVE-1 expression was detected only in a subpopulation comprising approximately 25 % of the MDI-treated pBM (Figure 3D-3E), which showed even higher expression levels of CD163 and CD206 (Figure 3F). Hence, LYVE-1^+^ macrophages form a distinct subpopulation in MDI-treated pBM which is more oriented towards an alternative phenotype.

### Macrophage-derived LYVE-1 is shedded by metalloproteinases

**Figure 4:**
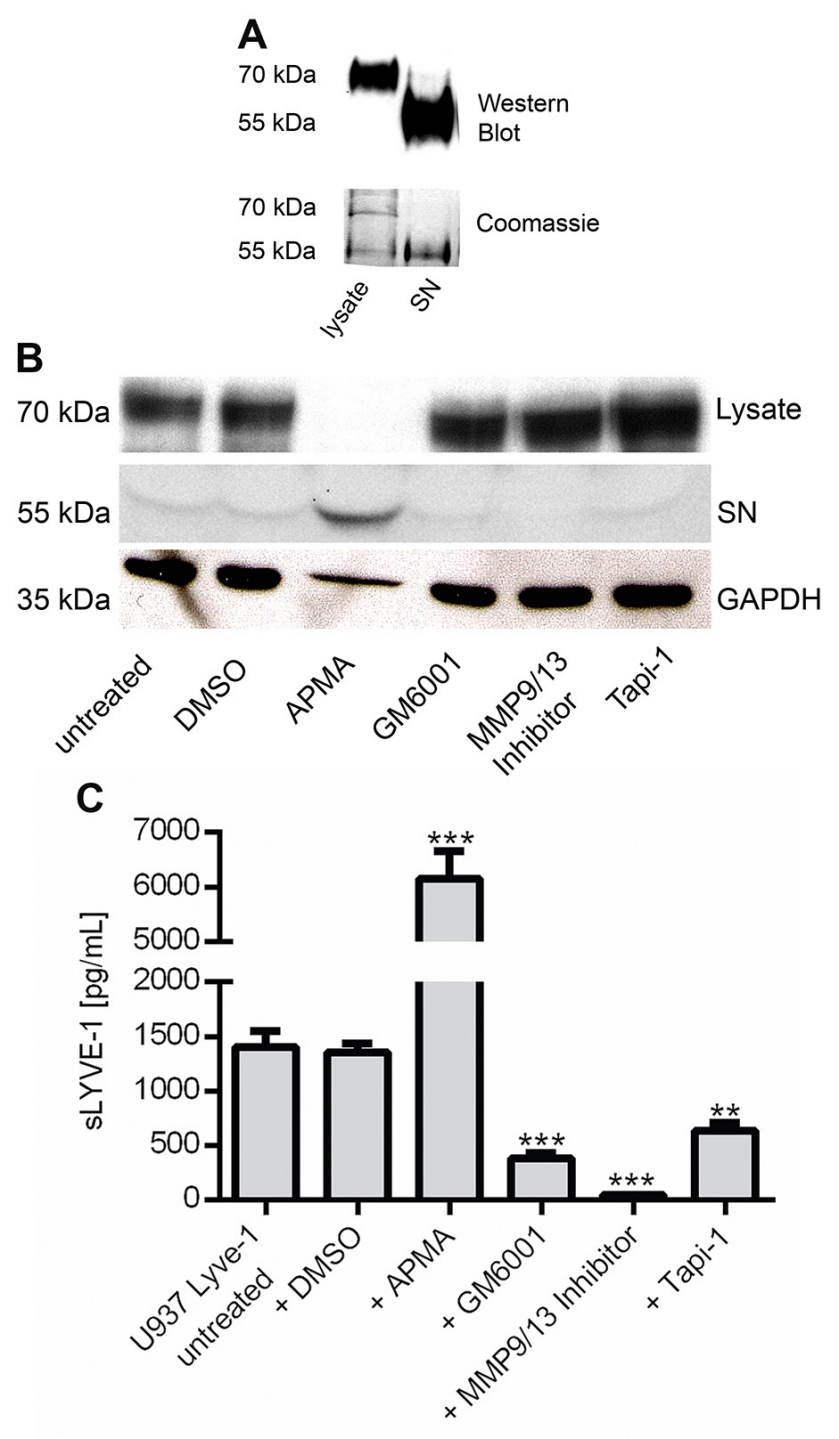
sLYVE-1 is shedded from macrophages by metalloproteinases **(A)** Western blot and Coomassie brilliant blue staining of U937 LYVE-1 cell lysates and supernatant (SN) after immunoprecipitation with a LYVE-1 antibody. **(B)** The transgenic cell line U937 LYVE-1 was stimulated for 24 h with shedding inducer 4-aminophenylmercuric acetate (APMA) and the inhibitors GM6001, MMP9/13 inhibitor and TAPI-1 (50 μM each), DMSO was added as a control. LYVE-1 was detected in total protein lysates and in the cell culture supernatant by western blot. **(C)** Quantification of sLYVE-1 by ELISA following treatment with different shedding modulators as indicated (n=3). Results are depicted as mean values with SEM.

It was not until recently that MT1-MMP and ADAM17 catalyzed shedding of LYVE-1 has been reported to occur in lymphatic endothelial cells [17, 18]. To investigate if LYVE-1 is shedded from macrophages in a similar manner, the human monocytic cell line U937 was lentivirally transduced with a *LYVE-1* cDNA containing expression vector. Detection of LYVE-1 after immunoprecipitation (IP) in western blot showed the full-length version (70 kDa) of the protein in the samples derived from U937 LYVE-1^+^ lysates, whereas an approximately 15 kDa shorter version was isolated from the cell culture supernatant (SN) (Figure 4A). Stimulation of the transgenic cell lines with the shedding activator 4-aminophenylmercuric acetate (APMA) resulted in the accumulation of sLYVE-1 in the supernatant. Stimulation with the shedding inhibitors GM6001, MMP9/13 Inhibitor or Tapi-1 led to a reduction of LYVE-1 shedding such that increased amounts of full-length LYVE-1 were detectable in the cell lysates (Figure 4B). Quantification of sLYVE-1 by ELISA in the supernatants of U937 LYVE-1^+^ cells following stimulation with the shedding modulators confirmed the western blot finding revealing that the MMP-9/13 inhibitor had the strongest inhibitory effect (Figure 4C). Besides MMP-9 and MMP-13, this small molecule inhibitor also targets metalloproteinases 1, 3 and 7. Hence, Lyve-1 secretion in macrophages occurs via a shedding process that is orchestrated by metalloproteinases identifying LYVE-1 as a substrate not only of MT1-MMP and ADAM17.

### sLYVE-1 acts as a decoy receptor for LMW-HA

**Figure 5:**
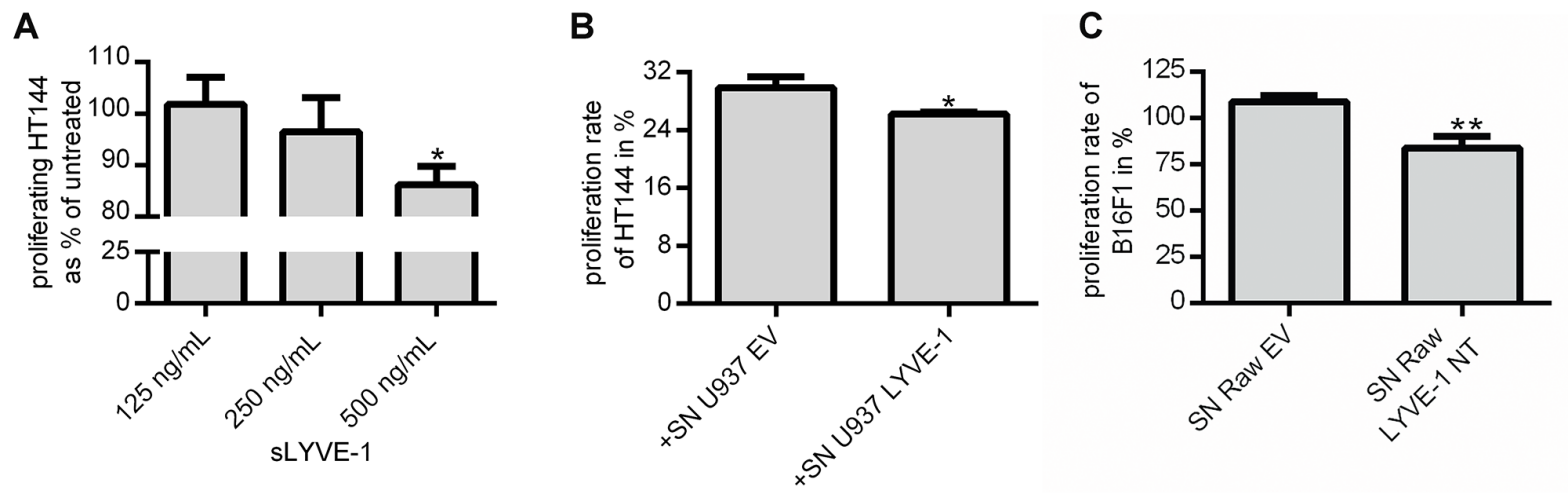
sLYVE-1 diminishes tumor cell proliferation by acting as a decoy receptor **(A)** Different concentrations of sLYVE-1 were added to HT144 cultured in medium supplemented with 2 % FCS. After 48h, the number of cells per well was determined by crystal violet staining in relation to untreated control cells (n=3). **(B)** Proliferation rate of HT144 was determined in a BrdU-based assay after culturing for 24 h in U937 EV/LYVE-1 conditioned FCS-free medium mixed 2:1 with fresh culture medium supplemented with 2 % FCS, (n=3). **(C)** B16F1 were seeded in low molecular weight-HA coated vessels and cultured for 48 h in conditioned medium derived from mock transfected Raw 264.7 cells (EV) or from cells expressing the LYVE-1 ectodomain (LYVE-1 NT) mixed 2:1 with fresh medium supplemented with 6 % FCS. Number of cells was determined by crystal violet staining in relation to untreated control cells (n=3). All data are mean values with SEM.

The high sequence homology of Lyve-1 and CD44 implies possible functional similarities between these HA-receptors at least to some extent. In a transplant model of malignant melanoma, the ectodomain of CD44 significantly reduced the tumor volume because of inhibition of tumor cell proliferation [19]. To test whether the shedded ectodomain of LYVE-1 also functions as a decoy receptor for the respective ligands such as HA, the proliferation rate of human and murine melanoma cell lines was evaluated after exposure to sLYVE-1 *in vitro*. Initially, the proliferation rate of the human melanoma cell line HT144 was assessed after treatment with synthesized sLYVE-1 showing that 500 ng/mL sLYVE-1 significantly inhibited melanoma cell proliferation (Figure 5A). In a similar manner, cultivation of HT144 in U937 conditioned medium resulted in a significant inhibition of proliferation of the human melanoma cell line specifically in medium derived from U937 LYVE-1^+^ cells (Figure 5B). Comparable results were also obtained using a murine model. It has been demonstrated previously that LMW-HA induces cell proliferation in B16F1 [20] (Supplementary Figure 3). The murine macrophage cell line RAW 264.7 was genetically engineered to express the ectodomain LYVE-1 NT. Conditioned medium from these cells was used for the cultivation of B16F1 in culture vessels which were coated with LMW-HA. Cellular proliferation was significantly inhibited when B16F1 were cultured in conditioned medium derived from Raw 264.7 Lyve-1 NT^+^ cells (Figure 5C).

The transgenic LYVE-1^+^ U937 and RAW264.7 cells did not show a higher proliferation rate, not even after HA stimulation (Supplementary Figure 2A-2D), although binding of HA to transgenic LYVE-1 could be confirmed in a binding assay (Supplementary Figure 4). These results together with the observed reduction of tumor growth in *Lyve-1* knockout mice support our postulated theory that sLYVE-1 acts as a decoy receptor for LMW-HA thereby inhibiting tumor cell proliferation *in vivo* and *in vitro*.

## DISCUSSION

TAMs, especially M2-like TAMs, have been described to exert important tumor-supportive roles. In melanoma, this is sustained by the finding that significantly increased numbers of TAMs correlate with greater invasiveness and the formation of metastasis [21]. Based on their tumor promoting functions TAMs have evolved as promising targets for adjuvant cancer therapies [22]. However, up to now, the use of pharmacological agents able to decrease TAM infiltration in the tumor stroma such as the colony stimulating factor 1 receptor inhibitor PLX3397 have not shown the expected results [23]. This could be due to a dual role played by TAM. Therefore, a thorough understanding of the functional properties of particular TAM subpopulations and their expressed molecules is required.

In this study we provide evidence, that the soluble form of Lyve-1 derived from macrophage-like cells significantly decreases melanoma cell proliferation by functioning as a decoy receptor for LMW-HA even though LYVE-1^+^ macrophages display a M2-like phenotype with a strong co-expression of the M2-markers CD206 and CD163. The M2-like phenotype of LYVE-1^+^ TAMs has already been described by others in TS/A tumors: Lyve-1 was differentially expressed in a subpopulation of macrophages displaying high expression of Arg-1, CD206, CD163 and Stab-1 [24].

In lymphatic endothelial cells membrane bound LYVE-1 was shown to promote LMW-HA-mediated endothelial cell proliferation. Since HA accumulates in the tumor interstitial fluid, the binding of LMW-HA to LYVE-1 on endothelial cells could thus promote tumor lymphangiogenesis and thereby the formation of metastases [25]. In our study, LMW-HA did not affect proliferation of LYVE-1^+^ human and murine macrophage-like cells. Also, sequential immunohistological staining of two TMAs showed significantly lower numbers of LYVE-1^+^ TAMs in higher melanoma stages, which points towards a reduced local amplification and/or recruitment of these cells. As each analyzed specimen on the TMAs was represented by two core sections, the TMAs were highly comparable to whole tissue sections [26].

The interesting finding in human melanomas instigated the B16F1 tumor experiment in mice. A higher tumor end weight accompanied by increased tumor cell proliferation was observed in Lyve-1^-/-^ mice pointing towards a tumor growth inhibiting effect of this molecule. In 2007, Gale et al. performed similar tumor growth experiments in Lyve-1^-/-^ mice using B16F10 melanoma cells. They did not notice any differences in tumor diameter after five weeks [16]. Accordingly, we observed tumor growth differences only in the early tumor growth phase, which vanished as soon as the tumors became necrotic and started ulcerating. Hence, sLYVE-1 dependent inhibition of melanoma cell proliferation influences predominantly early tumor growth; after that other tumor promoting factors seem to overrule the tumor-inhibiting effect of sLYVE-1.

To evaluate, whether LYVE-1^+^ TAMs are involved in angiogenic processes in hypoxic regions of the tumor, wild type B16F1 tumors were stained and analyzed. LYVE-1^+^ TAMs were located primarily in the marginal zone and not in central hypoxic regions, which has also been described in orthotopically grown mammary tumors [27]. This aspect argues against a pro-angiogenic role of these macrophages as they seem to be actively kept out of the tumor stroma.

In analogy to CD44, we showed that LYVE-1 in macrophages undergoes proteolytic cleavage leading to the shedding of its ectodomain mediated by metalloproteinases. Independent from our observations evidence for LYVE-1 shedding in lymphatic endothelial cells came from two further studies which identified LYVE-1 as a substrate of MT1-MMP and ADAM17 [17, 18]. MMPs and ADAMs are up-regulated in several tumors and have been associated with increased invasiveness and a shortened patient survival [28, 29]. Extensive shedding of LYVE-1 in the tumor microenvironment is, therefore, conceivable. Our results demonstrate that macrophage-derived sLYVE-1 inhibits the proliferation of tumor cells by acting as a decoy receptor for LMW-HA, which induces proliferation in melanoma cells mainly via interactions with CD44. As in Lyve-1^-/-^ mice, the proliferation rate of the melanoma cells was increased; we are convinced that excessive production of sLYVE-1 from macrophages and lymphatic endothelial cells in wild-type mice is the reason for impaired early tumor growth.

Concluding, we could show for the first time that sLYVE-1 exerts a tumor growth inhibiting function most probably by working as a decoy receptor for LMW-HA. The expression of a molecule with obvious anti-tumoral functions in M2-like TAM highlights the necessity to determine the net effect certain TAM subpopulations have on tumor growth before developing targeted therapies against these stroma cells.

## MATERIAL AND METHODS

### Mice

C57BL/6 wild-type, B6.129S1-Lyve1tm1Lhua/J (*LYVE-1*^-/-^) were purchased from The Jackson Laboratory. All mice were housed under specific pathogen-free conditions in the animal facility Mannheim. For tumor experiments only female mice between 8-12 weeks of age were used. Animal experimental protocols were approved by the animal ethics committee (Regierungspräsidium Karlsruhe, reference number: G42-14).

### Human samples

The study was performed in accordance to federal laws and regulations and institutional policies. We obtained ethical approval from the local ethical committee (reference number: 2010-318N-MA). Written informed consent was obtained from all patients and data was analyzed anonymously.

### Cells

U937, HT144 and B16F10 LUC cells were cultured in RPMI-1640 Medium (Thermo Fisher Scientific) supplemented with 10 % fetal calf serum (FCS), 100 U penicillin, as well as 100 mg/L streptomycin (Pen/Strep). Raw 264.7 and B16F1 cells were cultivated in DMEM (Thermo Fisher Scientific) with 10 % FCS, 1 % Pen/Strep. HEK 293T/17 cells were maintained in DMEM with 10 % FCS, 1 % Pen/Strep plus 100 mM sodium-pyruvate. Cell lines (all purchased from ATCC, B16F10 LUC from Bioware) were cultured at 37°C in an atmosphere enriched with 5 % CO_2_.

### Isolation of human peripheral blood monocytes (pBM)

CD14^+^ cells were isolated from buffy coats from healthy donors obtained from Red Cross Blood Service, Baden-Württemberg. In brief, after a gradient centrifugation using Biocoll (Merck Millipore), monocytes were isolated by magnetic-activated cell sorting via labeling with anti-CD14 microbeads (Miltenyi). 1x 10^6^ cells/mL were seeded in X-VIVO™ 15 medium (Lonza) for up to seven days at 37°C and 7.5% CO_2_. pBM were stimulated with M-CSF (100 ng/mL, Peprotech), IL-4 (10 ng/mL, Peprotech), dexamethasone (1x 10^-7^ M; 1000 U/mL, Sigma-Aldrich, St. Louis, MO, USA), GM-CSF (100 ng/mL, Peprotech), IFN-γ (10 ng/mL), IL-1β (10 ng/mL, Peprotech) and LPS (1 μg/mL, Invitrogen) as indicated.

### Immunohistochemistry

Cryostat sections (7 μm) from murine B16F1 tumors were air-dried and acetone-fixed. Cytospins of pBM were PFA-fixed. Subsequently, specimens were incubated with 0.3 % peroxide, 2 % BSA and the primary antibody. After incubation with the appropriate HRP-labelled secondary antibody, AEC^+^ chromogen solution (Biozol) was applied for visualization. Mayer’s Haemalaun (Merck) was used for counterstaining. Pictures were taken with a Leica DCRE microscope, Leica DC500 camera, and software system (Leica). Antibodies are listed in Supplementary Table 2.

### Sequential staining

Paraffin-embedded tissue samples were dewaxed and treated for 5 min with proteinase K (Dako) for antigen retrieval. After blocking with 5 % skimmed milk in Tris-buffered saline (TBS), the primary antibody was applied. Before the incubation with the HRP-labelled secondary antibody, specimens were treated with peroxidase blocking solution (Agilent). For detection VECTOR NovaRED Peroxidase solution (Vector Laboratories) was used. Counterstaining was performed with 10 % Haemalaun-solution. The specimens were placed successively into 80 %, 96 %, and 100 % ethanol and finally in xylene before being mounted with Eukitt (Kindler). After photodocumentation, the cover slips and mounting medium were removed by placing the samples into xylene overnight. Sections were re-hydrated by descending xylene/alcohol series, de-stained in stripping buffer (2 % SDS, 0.8 % β-mercaptoethanol, 62.5 mM Tris-HCl pH=7.5) at 50°C for 1 h and washed consecutively in water, 95 % ethanol, water, and TBS. Antibodies are listed in Supplementary Table 2.

### Tumor models

A total of 1x10^6^ B16F1 or B16F10 LUC cells were injected subcutaneously into the flank of 8-10 weeks old female mice. After ten days of tumor growth, mice were sacrificed. The tumors were weighed and snap frozen in liquid nitrogen. To monitor tumor growth *in vivo*, 150 μL of D-Luciferin potassium salt (Biovision) [30 mg/mL in PBS] was injected intraperitoneally into B16F10 LUC tumor-bearing mice. Luminescence pictures were taken 3 min after substrate injection with IVIS Lumina (PerkinElmer, Living Image 4.3).

### FACS analysis

1x10^6^ cells were prepared as a single cell suspension in PBS+0.1 % BSA. After treatment with Fc block (BD Bioscience), cells were stained with fluorochrome coupled antibodies. Fluorescence intensity was measured with FACS-Canto™II (BD Biosciences) and data were analyzed using FlowJO V10.1 software. Antibodies are listed in Supplementary Table 2.

### RT-PCR and qRT-PCR analysis

For RNA extraction innuPREP RNA Kit (Analytik Jena) was utilized. cDNA synthesis was performed using 1 μg RNA for reverse transcription with Maxima Reverse Transcriptase (Thermo Fisher Scientific) and Oligo (dt)_18_ primer (Thermo Fisher Scientific). For qRT-PCR, template cDNA was amplified with SyBRGreen Master Mix (Thermo Fisher Scientific) under standard conditions with an MX3000P sequence detection system (Stratagene). For normalization of the template amount gene expression was calculated in relation to the housekeeping gene β*-ACTIN.* Primers are listed in Supplementary Table 1.

### Western blot and immunoprecipitation (IP)

Cells were lysed with RIPA-P buffer. Proteins were separated using 10 % SDS-polyacrylamide gels. After semi-dry blotting (Bio-Rad) onto PVDF membranes (GE Healthcare) and blocking, the blots were incubated with primary antibodies overnight at 4°C. Subsequently, blots were incubated with secondary antibodies, and for signal detection, SuperSignal West Pico Chemiluminescent Substrate (Thermo Fisher Scientific) was used. Signal detection was performed with high-performance chemiluminescence films (GE Healthcare) and Curix 60 autoprocessor (Agfam). For IP experiments, U937 Lyve-1 cells were cultured in FCS-free medium overnight. Lysates were prepared using DISC buffer. 1 μg anti-Lyve-1 biotin antibody (R&D systems) and 40 μl Protein G-agarose (Sigma-Aldrich) were used per 4 mg protein. The mixture was incubated overnight at 4°C. After washing, the samples were boiled in 1x Laemmli buffer and separated on SDS-polyacrylamide gels and subjected to analysis by Western blot or Coomassie staining. Antibodies are listed in Supplementary Table 2.

### Generation of cell conditioned medium

1x 10^6^ cells/mL LYVE-1^+^ U937 or Raw 264.7 cells or the corresponding control cells were seeded in a FCS-free medium. After 24 h the supernatant was collected and centrifuged at 14,000 g for 10 min to obtain cell-free conditioned medium.

### Proliferation assay

HT144 cells were cultured for 24 h in U937 cells conditioned medium mixed 2:1 with fresh FCS-free medium. The cells were pulsed with 25 μg/mL BrdU (Sigma-Aldrich). BrdU-labeled cells were detected by flow cytometry using an anti-BrdU-FITC antibody. HT144 cells were seeded in RPMI supplemented with 2 % FCS and incubated for 2 h. Subsequently, sLYVE-1 (R&D Systems) was added at different concentrations for 48 h. Coating of 96-well plates was performed as described previously [30]. In brief, culture vessels were incubated overnight with 1 mg/mL LMW-HA (15-40 kDa, *S.pyogenes* fermentation, R&D Systems) in 50 mM NaHCO_3_ buffer at 4°C. After extensive washing with PBS, 3x 10^3^ B16F1 cells were seeded in 100 μL DMEM supplemented with 6 % FCS, 200 μL of conditioned medium derived from LYVE-1^+^ and mock transfected U937 or Raw 264.7 cells. The cells were fixed and stained with 5 % crystal violet (Roth) in 20 % methanol (Sigma-Aldrich) and air-dried. Stained cells were dissolved in 100 % methanol and quantified with Tecan Microplate Reader (Tecan Group) at λ=570 nm.

### Generation of transgenic cell lines

Human LYVE-1 cDNA (clone IRAUp969G0386D, SourceBioscience) or murine LYVE-1 cDNA (clone IRAVp968E0743D, SourceBioscience) was amplified by PCR and cloned into a modified lentiviral expression system vector pHAGE [31]. The ectodomain of murine Lyve-1 was generated by PCR-based amplification of the nucleotides 1 to 703 of the Lyve-1 cDNA. HEK293/T cells were transfected with 3^rd^ generation lentiviral plasmids (pMD2.G L1, pRSV rev L2, pMDLg/pRRE L3 and pCDNA3.1/p35 E 71) in combination with the transfer modified pHAGE empty vector (EV) or pHAGE-Lyve-1. U937 and Raw 264.7 cells were infected with generated lentiviruses, and transduced cells were selected by resistance to puromycin (2 μg/mL, Thermo Fisher Scientific). Primers are listed in Supplementary Table 1.

### Statistics

Data were statistically evaluated with GraphPad Prism 6.0 (GraphPad Software, USA). Statistics were made using standard Student’s t-test or one-way ANOVA and Bonferroni correction. The level of significance is indicated by asterisks (***≤0.001; **≤0.01 and *≤0.05). Error bars show SEM of each experiment.

## SUPPLEMENTARY MATERIALS FIGURES AND TABLES



## Abbreviations

ADAM: (a disintegrin and metalloproteinase)
APMA: (4-aminophenylmercuric acetate)
BMDM: (bone marrow-derived macrophages)
dexa: (dexamethasone)
GR: (glucocorticoid receptor)
HMW-HA: (high-molecular weight hyaluronic acid)
IL: (interleukin)
IP: (immunoprecipitation)
LMW-HA: (Low-molecular weight hyaluronic acid)
LYVE-1: (lymphatic vessel endothelial hyaluronan receptor-1)
MAPK: (mitogen-activated protein kinase)
M-CSF: (macrophage colony-stimulating factor)
MDI: (M-CSF/dexa/IL-4)
MF: (mifepristone)
MT-MMP: (membrane-type metalloproteinase)
pBM: (peripheral blood monocytes)
sLYVE-1: (soluble Lyve-1)
SN: (supernatant)
TAM: (tumor-associated macrophages)
