# Supplementary material for: The shedded ectodomain of Lyve-1 expressed on M2-like tumor-associated macrophages inhibits melanoma cell proliferation
Source: Oncotarget. 2017 Oct 10;8(61):103682–92. doi: 10.18632/oncotarget.21771 (PMC5732759; doi:10.18632/oncotarget.21771)
Supplement: Supplementary file 1 [file oncotarget-08-103682-s001.pdf]

# The shedded ectodomain of Lyve-1 expressed on M2-like tumor-associated macrophages inhibits melanoma cell proliferation

## SUPPLEMENTARY MATERIALS

### Supplementary methods

#### BrdU-based proliferation assay

U937 EV/Lyve-1 were seeded in medium containing 0.2% FCS and allowed to adapt to the culture conditions for 4 h. Transgene U937 or Raw 264.7 were treated with 10 µg/mL LMW-HA (R&D Systems, 15-40 kDa) or 10 µg/mL HMW-HA (R&D Systems, >950 kDa) overnight. The cells were pulsed with 25µg/mL BrdU (Sigma). BrDU labelled cells were detected by flow cytometry using an anti-BrDU-FITC antibody.

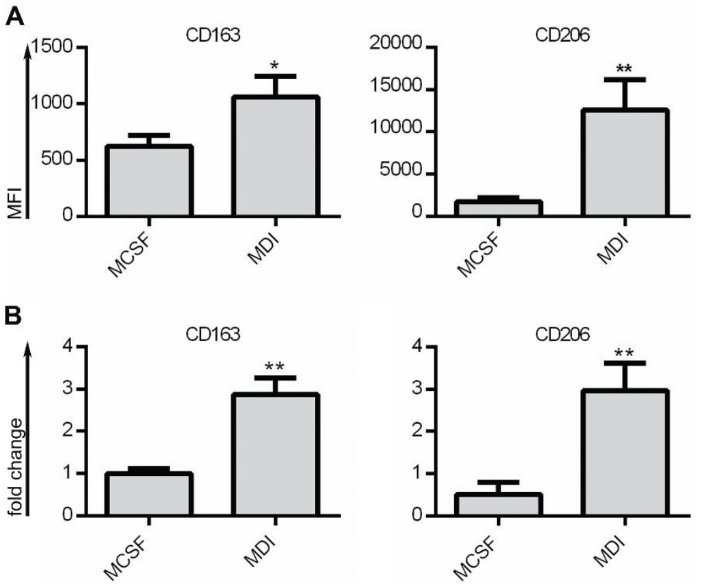


**Supplementary Figure 1: M-CSF/dexa/IL-4 induces up-regulation of CD163 and CD206 in pBM.** pBM were stimulated for seven days with M-CSF or M-CSF/dexa/IL4 (MDI) as indicated. **(A)** Comparison of protein expression levels by assessment of the median fluorescence intensities (MFI) by FACS, n=10. **(B)** qRT-PCR analysis determined mRNA expression relative to β-*ACTIN* mRNA levels. MDI values are given as fold-induction over expression levels of M-CSF treated control, (n=6). Results are depicted as mean values with SEM.

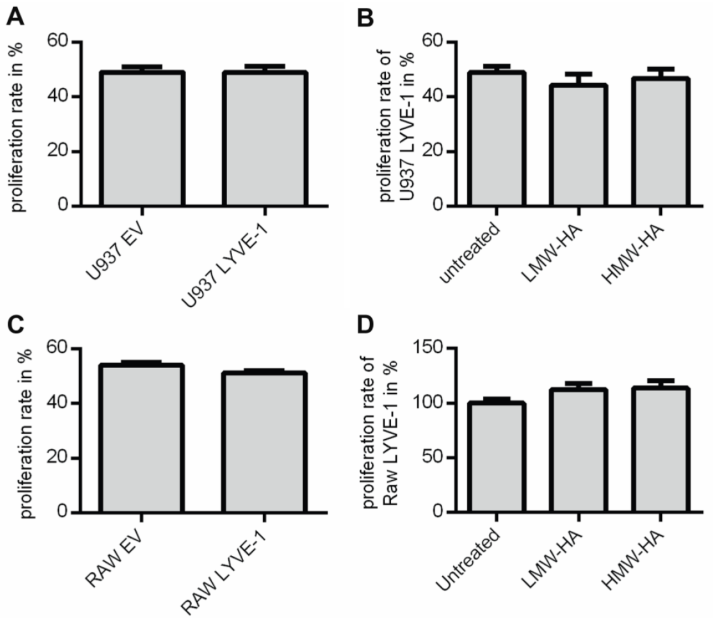


**Supplementary Figure 2: Overexpression of LYVE-1 does not affect the proliferation rate of transgene U937 and RAW264.7. (A, C)** Proliferation rate of transgene, BrDU-pulsed U937 and Raw 264.7 after culturing for 24 h in medium supplemented with 0.2 % FCS, n=5. **(B, D)** EV and LYVE-1 transgenic cell lines were stimulated overnight with HA as indicated in culture medium containing 0.2 % FCS and the proliferation rate was assessed, (n=5).

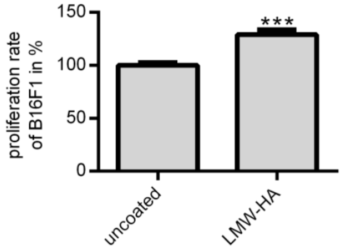


**Supplementary Figure 3: LMW-HA induces proliferation of B16F1 melanoma cells.** B16F1 were seeded in vessels which were either coated with 1 mg/mL LMW-HA or untreated in medium containing 2 % FCS. Number of cells was determined by crystal violet staining in relation to untreated control cells (n=3).

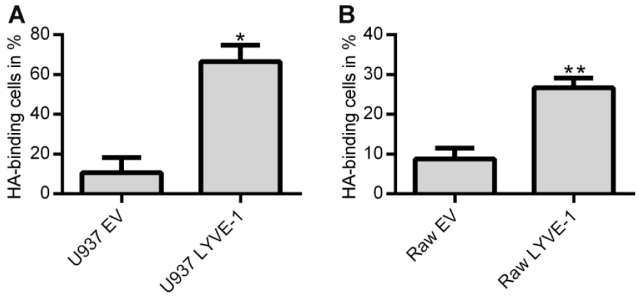


**Supplementary Figure 4: HA-binding to LYVE-1$^+$ U937 and LYVE-1$^+$ RAW264.7 is enhanced.** Ligand binding capability of **(A)** LYVE-1$^+$ U937 and **(B)** RAW264.7 cells was examined by incubation with biotinylated HA. Percentage of HA-binding cells was determined by flow cytometry using strepta-FITC to detect bound HA, (n=3).

**Supplementary Table 1: Primer Sequences**

| CLONING | SEQUENCE |
|---|---|
| HS LYVE-1 XbaI Fw | GAT TCT AGA CAC GAT GGC CAG GTG CTT |
| HS LYVE-1 MluI Rev | GAT ACG CGT CTA AAC TTC AGC TTC CAG GCA |
| MM LYVE-1 XbaI Fw | TTA TCT AGA GGG ATC TGC ACA ATG CT |
| MM LYVE-1 MluI Rev | AAT ACG CGT TGC ATC TAA ACT TCA GCT T |
| MM LYVE-1 XbaI Fw | TTA TCT AGA GGG ATC TGC ACA ATG CT |
| MM LYVE-1 MluI Rev | GAT CAC GCG TGC GGT GGG GAC ACC TCC A |
| **qRT-PCR** | **SEQUENCE** |
| HS CD163 Fw | GCC ACA ACA GGT CGC TCA TCC |
| HS CD163 Rev | GTG TGG CTC AGA ATG GCC TCC |
| HS CD206 Fw | TGG TTT CCA TTG AAA GTG CTG C |
| HS CD206 Rev | TTC CTG GGC TTG ACT GAC TGT TA |
| HS ACTIN Fw | GGC ACC ACA CCT TCT ACA ATG A |
| HS ACTIN Rev | TCT CCT TAA TGT CAC GCA CGA T |
| HS LYVE-1 Fw | CTT GCA GCT ATG GCT GGG TT |
| HS LYVE-1 Rev | TAA GGG GAT GCC ACC GAG TA |

**Supplementary Table 2: Antibodies**

| 1st ANTIBODY | APPLICATION | COMPANY |
|---|---|---|
| PE-Cy 5 anti-hs CD206 | FACS | BD Bioscience (#551136) |
| biotin anti-hs Lyve-1 | Western Blot / IP | R & D (BAF2089) |
| anti-hs Lyve-1 | IHC (PFA Fixation, Antigen retrieval Proteinase K) | Abcam (ab 36993) |
| anti-hs CD68 | IHC (Antigen retrieval Proteinase K) | Dako (M087629-2) |
| anit-mm / hs GAPDH | Western Blot | Santa Cruz (SC25778) |
| anti-mm CD68 | IHC / IF (Acetone Fixation) | ABD (MCS1957) |
| anti-mm CD31 | IHC / IF (Acetone Fixation) | BD Bioscience (#550274) |
| anti-mm/hs Ki67 | IHC / IF (PFA Fixation) | Abcam (ab16667) |
